# The Rod Steers the Globe in ATG9A-Mediated Lipid Transport

**DOI:** 10.1080/27694127.2024.2360356

**Published:** 2024-05-30

**Authors:** Alexander R. van Vliet, Sharon A. Tooze

**Affiliations:** a Molecular Cell Biology of Autophagy, The Francis Crick Institute, London NW1 1AT, UK; b Present Address: MRC Laboratory of Molecular Biology, Cambridge CB2 0QH, UK

List of abbreviations:

ACSL, acyl-CoA synthetase long chain family member; ATG, autophagy-related; ATG2A, autophagy-related 2A; ATG9A, autophagy-related 9A; IP, immunoprecipitation; RBG: repeating beta groove; VPS13, vacuolar protein sorting 13

ATG9A (autophagy-related 9A), and the related protein ATG9B, are the only multi-spanning membrane proteins amongst the autophagy related (ATG) protein cohort first identified in yeast and subsequently mammalian cells (about 40 in yeast). Given this topology it was immediately recognized it would play a critical role in early stages of autophagy, including phagophore (isolation membrane) and autophagosome biogenesis. Work over the last few years on ATG9A has uncovered a substantial piece of the puzzle regarding autophagosome formation, showing that one of the functions of ATG9A is to act as a lipid scramblase. Our previous work, combined with others, has shown that this scramblase function is physically connected to the lipid transfer function of ATG2A (autophagy-related 2A) proteins: the two proteins forming a lipid transfer complex at the centre of autophagic membrane expansion.

In our recent study [1], in order to gain a better understanding of ATG9A’s interactor network, we performed an endogenous immunoprecipitation (IP) using two different detergents. Our own observations had indicated that ATG9A overexpression could lead to localization and aggregation artefacts, and so we surmised that an endogenous IP followed by mass spectrometry offered the best strategy to gain new insights into ATG9A’s interactor network. Importantly, the successful purification and structural determination of ATG9A and other transmembrane proteins in recent years has relied on a specific class of detergents distinct from classical non-ionic detergents like Triton-X100. These detergents, including the maltose neopentyl glycol detergents represented by lauryl maltose neopentyl glycol (LMNG) and the alkyl maltosides like n-dodecyl-β-d-maltopyranoside (DDM), better preserve the native conformation of hydrophobic proteins. This success prompted us to utilize these detergents in our experiments. We hypothesized that this strategy would be more effective in maintaining the native state of ATG9A, thereby enhancing the preservation of its interactors within the cell and giving a different set of interactors than solubilization using Triton-X100.

Our interactome study identified intriguing new interactors related to lipid metabolism, including ACSL1 (acyl-CoA synthetase long chain family member 1) and ACSL3, and VPS13A (vacuolar protein sorting 13 homolog A) and VPS13C. The presence of ACSL1 and ACSL3, enzymes responsible for activating fatty acids, is particularly intriguing as these account for crucial steps in phospholipid synthesis, a process increasingly linked to autophagosome biogenesis. While our study did not definitively demonstrate a direct interaction between ATG9A and ACSL proteins, we speculate they might be part of a larger ATG9A complex, potentially involving ATG2 or VPS13 proteins. This hypothesis is also consistent with the recent paper of the Martens lab (MFPL, Vienna, Austria) on Atg9 and its association with Faa1. This interaction could facilitate phospholipid mobilization and supply for autophagosome formation, in agreement with previous work by the Graef lab (Cornell University, Cornell, USA) where de novo phospholipid synthesis was shown to be important for autophagosome formation. Our own previous work characterized the complex between ATG9A and ATG2A, and so we were most interested in the presence of VPS13A and VPS13C in our IP dataset, as these proteins are all part of a new class of lipid transport proteins termed the repeating beta groove (RBG) superfamily. Additionally, a recent study from the Graef lab demonstrated the involvement of Vps13 in autophagy in yeast, further highlighting their potential importance in relation to ATG9A. The RBG family of proteins have been shown to bind two separate membranes through domains on their N- and C- termini, with a hydrophobic groove running along the length of the RBG domain, allowing the hydrophobic tail of phospholipids to be shielded from the hydrophilic cytosol.

We found that VPS13A is able to directly bind to ATG9A. Interestingly, our co-IP data using VPS13A fragments suggests that both the N- and C-termini of VPS13A could bind ATG9A, contrasting with our previous work where only C-terminal ATG2A binding was observed. However, limitations in expressing the full VPS13A RBG domain might have skewed the results towards N-terminal binding. VPS13A has a FFAT motif (two phenylalanines (FF) in an acidic tract) at its N-terminus, with which it binds Vesicle-associated membrane protein (VAMP) Associated Proteins (VAPs) at the ER. Whether any N-terminal binding to ATG9A competes with the FFAT motif for ER binding is not clear and further investigation is needed to clarify the interaction and its potential competition. Using colabfold we generated the structure for full length VPS13A. We were especially interested in the predicted structure of the C-terminus as we wanted to determine the binding site of ATG9A in more detail. Analysis of the C-terminus of VPS13A showed the presence of four alpha helical domains. We systematically deleted each helix and identified one (termed helix B in the study) as the putative ATG9A binding site. In order to try and uncover a physiological role for the ATG9A:VPS13A complex, we relied on the lipid droplet binding property of the C-terminus of VPS13A, as shown by the De Camilli lab (Yale University, New Haven, USA). When we expressed this fragment in cells fed with oleic acid, we could see an enrichment around lipid droplets. When imaging ATG9A-positive vesicles in these cells, we found that these also enriched around lipid droplets, but only in cells expressing the VPS13A construct. To further validate this observation, we employed correlative light and electron microscopy and were able to visualize vesicular/tubular structures that correlated well with ATG9A fluorescence.

As the C-terminus of ATG2A is required for autophagy, and the VPS13A C-terminus shares homology with the ATG2A C-terminus, we developed an ATG2A-VPS13A chimera to test if the ATG2A C-terminus has a unique functional significance in autophagy. Surprisingly, this chimera, despite retaining its ability to interact with ATG9A as efficiently as wild-type ATG2A, failed to fully restore autophagic flux in ATG2A/B knockout cells. This result can be interpreted in a variety of ways: One of these suggests that, beyond ATG9A binding, the C-terminus of ATG2A might harbour essential structural characteristics that are absent in the VPS13A counterpart. It is also possible that the chimeric C-terminus prevents the correct (functional) formation of the ATG9A:ATG2A complex.

VPS13A and VPS13C have been established to mediate lipid trafficking between organelles such as lipid droplets, mitochondria, lysosomes and the ER. These diverse organelle-specific activities support a role for the ATG9A:VPS13A complex outside of autophagy and in mediating lipid trafficking more generally. Our data on the recruitment of ATG9A to lipid droplets, in combination with previous results from the Bonifacino lab (NIH, Bethesda, USA), supports this possibility. Future work could expand on this finding to explore a role for ATG9A-positive vesicles as a mobile lipid repository capable of shuttling lipids dynamically between different organelles.
